# Prevalence and Correlates of Muscle-Strengthening Activity Participation in Croatia: A Cross-Sectional Study in a National Representative Sample of 4561 Adults

**DOI:** 10.3390/ijerph18178905

**Published:** 2021-08-24

**Authors:** Hrvoje Radašević, Jelena Čvrljak, Željko Pedišić, Danijel Jurakić

**Affiliations:** 1 Andrija Stampar Teaching Institute of Public Health, 10000 Zagreb, Croatia; hrvoje.radasevic@stampar.hr (H.R.); jelena.cvrljak@stampar.hr (J.Č.); 2 Institute for Health and Sport, Victoria University, Melbourne, VIC 80001, Australia; 3 Faculty of Kinesiology, University of Zagreb, 10000 Zagreb, Croatia; danijel.jurakic@kif.unizg.hr

**Keywords:** physical inactivity, motor activity, exercise, resistance training, surveillance

## Abstract

The World Health Organization recommends adults to engage in muscle-strengthening activity (MSA) at least two times per week. The aim of this study was to determine the prevalence and correlates of MSA in Croatian adults. We analysed self-reported data collected among 4561 Croatians aged ≥18 years within the European Health Interview Survey (EHIS wave 2). We calculated the weighted prevalence of meeting the MSA guidelines, and odds ratios for different population groups, adjusted for a range of sociodemographic and lifestyle variables in a multivariable logistic regression analysis. The prevalence of meeting the MSA guidelines was 8.0% (95% CI: 7.2, 8.8) in the overall sample, 5.4% (95% CI: 4.5, 6.4) among females, and 10.9% (95% CI: 9.6, 12.3) among males. We found significantly lower odds of meeting the MSA guidelines for females, older age groups, inhabitants of sparsely populated areas, those with a low education level, obese individuals, and those who did not rate their health as “very good” (*p* < 0.05 for all). The vast majority of Croatian adults do not meet the MSA guidelines. Public health initiatives to promote MSA in Croatia should focus on females, seniors, sparsely populated areas, people with low education, obese individuals, and those with impaired health.

## 1. Introduction

Non-communicable diseases (NCDs) are among the most prevalent illnesses and are the leading cause of mortality worldwide [1]. It was estimated that physical inactivity is responsible for 6% to 10% of deaths from NCDs [2]. According to the World Health Organization (WHO)’s estimates, physical inactivity is the fourth highest ranked mortality risk factor globally [3]. Epidemiological studies have shown that physical activity can reduce the risk of a number of chronic diseases and conditions, such as coronary heart disease [4], hypertension [5], metabolic syndrome [6], and cancer [7]. Increased physical activity at the population level could therefore significantly improve population health [2].

Although physical activity guidelines largely focus on the benefits of aerobic physical activity, they also include recommendations for muscle-strengthening activities (MSA) [8,9,10]. MSA are physical activities that are performed to increase the strength, power, endurance, and/or mass of skeletal muscles [11]. Usually, they are part of an exercise routine and involve the use of hand-held weights, weight machines, exercise bands, or the individual’s own body weight (e.g., non-weight-bearing squats or push-ups) [12]. According to the current WHO physical activity guidelines, adults (18–64 years) and older adults (≥ 65 years) should participate in MSA on two or more days a week [8]. In the recently proposed “Croatian 24-H Guidelines for Physical Activity, Sedentary Behaviour, and Sleep”, adults and older adults are encouraged to engage in MSA at least two times a week [13].

Numerous studies have shown the health benefits of MSA, such as improvements in strength [14], physical performance [15], and physical functioning [16]. MSA may also help in the prevention of cardiovascular disease [17], prevention and treatment of type 2 diabetes [18], prevention or delay of obesity [19], preservation of bone and muscle mass [20], and the improvement of mental health [21]. Ciolac and Rodrigues da Silva [22] stated that MSA may be an effective treatment for rheumatoid arthritis, osteoarthritis, and low back pain, while Saeidifard et al. [23] found an association of MSA with lower mortality risk, especially if it is combined with aerobic exercise.

Studies on the prevalence and correlates of MSA have been conducted in several countries, such as Australia [24,25,26], the European Union member states [27], Finland [28], Japan [29], Korea [30], Scotland [31], and the United States [32,33,34]. These studies showed a varying prevalence of adults meeting the MSA guidelines, ranging between 0.7% in Romania and 51.6% in Iceland [27]. A recent systematic review on correlates of MSA included 51 studies from nine countries [35] and found that MSA participation was low among those with poor self-rated health and low education levels. Self-efficacy, affective judgments, self-regulation behaviours, subjective norms, and programme leadership were identified as factors that may promote MSA [35]. Findings of individual studies on the association between MSA and a range of potential correlates, such as age, sex, race, income, and body mass index (BMI), were inconsistent [35].

There are no published data on the correlates of MSA in Croatia. Liangruenrom et al. [36] suggested that correlates of physical activity may be country-specific. Given that the findings of previous studies on MSA participation and a number of its correlates are inconsistent, the aim of this study was to determine the prevalence and correlates of MSA in a national representative sample of Croatian adults. We hypothesised that the prevalence of meeting the MSA guidelines is low, and that it would vary significantly across different population groups.

## 2. Materials and Methods

We used data from the European Health Interview Survey (EHIS), a standardised survey on health status, health care use, and determinants of health that is conducted in all European Union member states every five years. EHIS was conducted in Croatia for the first time as a part of its second cycle (EHIS wave 2). The EHIS wave 2 was conducted in Croatia from April 2014 to March 2015 by the Croatian Institute of Public Health, in cooperation with county-level institutes of public health, the Central Bureau of Statistics, and the Ministry of Health. A two-stage stratified random sample of private dwellings was used to ensure that the sample was representative of the Croatian population. A total of 3140 private households were included in the sample. Persons who lived in collective households (e.g., nursing homes, boarding schools, hospitals, monasteries) were not included in the survey. The sample included all individuals within the selected households who were at least 15 years old at the time of the survey. Sampling was done by the Central Bureau of Statistics, based on data from the 2011 Census of Population, Households and Dwellings. The Ethics Committee of the Croatian Institute of Public Health approved the study (number: 80-581/1-14; 27 March 2014) and all respondents signed an informed consent form before participating in the study. Trained surveyors conducted face-to-face interviews with the study participants. The total sample included 5446 participants aged 15 to 96 years. For the purpose of this study, we used data from 4561 participants aged 18–96 years, who responded to the question about MSA. More details regarding EHIS wave 2 methods can be found elsewhere [37,38]. Sample characteristics are presented in Table 1.

### 2.1. Measures

#### 2.1.1. Muscle-Strengthening Activity

The EHIS wave 2 questionnaire asked respondents about their participation in MSA using the question: “In a typical week, on how many days do you carry out physical activities specifically designed to strengthen your muscles, such as doing resistance training or strength exercises?” According to the proposed Croatian MSA guidelines [13], data were categorised as: (i) “meeting the MSA guidelines” (MSA participation on ≥2 days/week), or (ii) “not meeting the MSA guidelines” (MSA participation on <2 days/week).

#### 2.1.2. Correlates of Muscle-Strengthening Activity

Data on sex, age, urbanisation level, education level, self-perceived health, smoking status, alcohol intake, height, and weight were collected using the standard EHIS questionnaire [37]. Urbanisation level was presented in three categories: (1) Densely populated area; (2) Moderately populated area; and (3) Sparsely populated area. Education level was expressed using three categories: (1) High level of education (including professional, undergraduate, graduate, and postgraduate); (2) Medium level of education (including high school and vocational training after high school that does not include higher education); and (3) Low level of education (including preschool and elementary school education). Self-perceived health was assessed on the following scale: (1) Very good; (2) Good; (3) Fair; (4) Bad or very bad. Smoking status was presented in two categories: (1) Non-smokers; and (2) Smokers. Alcohol intake was assessed using the following question: “In the past 12 months, how often did you drink alcoholic beverages of any kind?”, with a 4-point response scale, ranging from: (1) Rarely or never to (4) ≥ 3 days/week. Self-reported height and weight data were used to calculate BMI. According to their BMI, participants were categorised into the following categories: <18.5 kg/m^2^ (underweight); from 18.5 kg/m^2^ to 24.9 kg/m^2^ (”normal” weight); from 25 kg/m^2^ to 29.9 kg/m^2^ (overweight); and ≥30 kg/m^2^ (obese).

### 2.2. Statistical Analysis

Data were analysed using IBM SPSS Statistics 25 (IBM Corp, Armonk, NY, USA). We calculated the prevalence of meeting MSA guidelines and its 95% confidence intervals (95% CI) for the overall sample and stratified by age, urbanisation level, education level, smoking status, alcohol intake, BMI categories, and self-perceived health. Multivariable logistic regression was used to analyse the associations between sociodemographic, health, and lifestyle-related variables and the meeting/not meeting of MSA guidelines. The analyses included the following explanatory variables: Sex (reference group [ref] = “males”); Age (ref = “18–24 years”); Urbanisation level (ref = “densely populated area”); Education level (ref = “high”); Self-perceived health (ref = “very good”); BMI (ref = “’normal weight”); Smoking status (ref = “non-smokers”); and Alcohol intake (ref = “rarely or never”). Adjusted odds ratios and their 95% CIs were presented. In all statistical tests, a *p*-value of < 0.05 was considered to indicate statistical significance. All analyses were conducted using sampling weights to ensure that the estimates were population representative. Missing data were imputed using the expectation–maximization algorithm [39].

## 3. Results

In the total sample, 8.0% (95% CI: 7.2, 8.7) of participants met the MSA guidelines (Table 2). Unadjusted analysis revealed significant differences in the prevalence of meeting MSE guidelines between the groups by sex, age, urbanisation level, education level, alcohol intake, BMI, and self-perceived health (*p* < 0.001) (Table 2). In the adjusted analysis, significant associations with meeting the MSA guidelines were found for sex, age, urbanisation level, education level, BMI, and self-perceived health (*p* < 0.05) (Table 3).

Specifically, females had 49% (95% CI: 34, 61) lower odds of meeting the MSA guidelines, compared with males. Compared with the youngest age group (18–24 years), other age groups had significantly lower odds of meeting the MSA guidelines, with adjusted odds ratios ranging from 0.49 (95% CI: 0.36, 0.68) for 25–39-year-olds to 0.18 (95% CI: 0.11, 0.31) for older adults (65+ years). Compared to the inhabitants of densely populated areas, those who lived in sparsely populated areas had 37% (95% CI: 16, 52) lower odds of meeting the MSA guidelines. The adjusted odds ratio for the inhabitants of medium populated areas was not significant. Compared with the “high education” group, the odds of meeting the MSA guidelines were 50% (95% CI: 36, 61) and 77% (95% CI: 62, 86) lower in the “medium education” and “low education” groups, respectively. Furthermore, compared with the group with “normal” weight (BMI from 18.5 to 24.9), the individuals classified as obese (BMI ≥ 30) had 45% (95% CI: 14, 64) lower odds of meeting the MSA guidelines. No significant associations with meeting the MSA guidelines were found for being “underweight” (BMI < 18.5) and “overweight” (BMI from 25 to 29.9). Furthermore, the highest odds of meeting the MSA guidelines were found for those who rated their health as “very good”. Compared to them, those who rated their health as “good”, “fair”, and “bad or very bad”, respectively, had 28% (95% CI: 7, 45), 40% (95% CI: 14, 59), and 63% (95% CI: 32, 80) lower odds of meeting the MSA guidelines.

For smoking status and alcohol intake, we found no significant associations with meeting the MSA guidelines (*p* > 0.05).

## 4. Discussion

In a large, population–representative sample, we found that the vast majority of Croatian adults do not meet the MSA guidelines. Lower odds of meeting the MSA guidelines were found for females, older age groups, inhabitants of sparsely populated areas, those with low education level, people with obesity, and those who did not rate their health as “very good”. No significant associations with meeting the MSA guidelines were found for smoking status and alcohol intake.

Given the positive effects of muscle-strengthening activities on health [15,17,18,19,20,21,40], from a public health perspective, the results of our study are concerning. The percentage of adults meeting the MSA guidelines in Croatia is higher than in Japan [29] and similar to that of Australia [24], Korea [30], and the United States [34,41]. However, Croatia is among the European Union countries with the lowest prevalence of meeting the guidelines [27]. Potential reasons for the low involvement of Croatian adults in MSA could be a low motivation for physical activity in general and a lack of interest for MSA in particular. The low prevalence of meeting the MSA guidelines in Croatia might also be due to inadequate access to muscle-strengthening exercise facilities, programmes, and equipment, as well as a lack of financial support for MSA programs and promotion provided by the national and local-level governments. According to Eurobarometer data [42], only 7% of people in Croatia exercise in health and fitness centres. According to the Central Bureau of Statistics, in 2019, there were only 294 registered fitness centres in Croatia, that is, 1 centre per 14,622 inhabitants [43]. Given the low prevalence of meeting the MSA guidelines and relatively low accessibility of facilities that enable such activities in Croatia, there is a need for public health actions to promote MSA. A potential solution for this issue is the promotion of MSA outside fitness centres; for example, at home. With regards to the muscle hypertrophy, MSA using one’s own body weight at home might be as effective as exercising with weights in a fitness centre. A recent meta-analysis did not find significant differences in the effects on muscle hypertrophy for exercising with lower loads and with heavy weights [44]. Additionally, fitness centres usually charge membership fees. One of the key perceived barriers for exercise and sport among Croatian adults [42] is that “it is too expensive”. This barrier could be circumvented by promoting home-based MSA.

Findings on sociodemographic correlates of MSA in the current study were largely consistent with findings of previous studies. In line with our findings, previous studies have shown that being a female, older age, and lower education level are associated with lower odds of participation in MSA [45]. The lower odds of meeting the MSA guidelines among Croatian females could be explained by their motives for exercise. The most important motives for exercise among Croatian females were relaxation and fitness improvement, while the least important motive was the muscle mass gain [46]. Physiological changes associated with aging, including those affecting skeletal muscles, have a large impact on physical fitness [47]. The lower level of participation of the elderly in MSA might be partially explained by the overall decline in their physical fitness and mobility due to the ageing process. Older people are more susceptible to injuries, which may be another factor that deters them from participating in MSA [48,49]. Furthermore, the individuals with a low level of education are likely to have less knowledge about the health benefits of physical activity, compared with highly educated people [50]. It might be that the awareness of the importance of MSA is also lower among those with a low level of education, which would explain the lower odds of meeting the MSA guidelines in this population group. Furthermore, living in a sparsely populated area was negatively associated with MSA participation. This might be due to lower accessibility to facilities that offer muscle-strengthening exercise programmes in rural areas, compared with urban areas.

Our findings are also in line with previous studies showing a lower prevalence of MSA among obese individuals [41,51,52] and among those with poor self-rated health [28,53,54]. The lower participation in MSA among obese individuals might partially be explained by the fact that recommendations for the prevention and treatment of obesity are mainly focused on aerobic physical activities [19]. In light of our findings, and given that MSA may significantly contribute to achieving a negative energy balance [15,19], it would be particularly important to target obese individuals in future MSA-promoting initiatives in Croatia. Furthermore, poor health may discourage or prevent people from participating in strenuous exercise. This may explain why the lowest participation in MSA was associated with people who rated their overall health as bad or very bad.

The key strengths of this study were the use of a large, nationally-representative sample of Croatian adults, which allowed us to make inferences about the population, and the use of standardised EHIS questionnaire, which allowed us to make comparisons with findings of EHIS surveys conducted in other European countries.

The key limitation of the study stems from its cross-sectional design; we were not able to draw conclusions about the direction of the relationships. Some of the relationships found in our study may be bidirectional. For example, it may be that people with poor health were less likely to engage in MSA, but it may also be that participation in MSA improved health of some participants. Future longitudinal studies on this topic are needed to elucidate the direction of the relationship.

## 5. Conclusions

We found that the vast majority of Croatian adults do not meet the MSA guidelines, and that lower odds of meeting the MSA guidelines are associated with female sex, older age groups, living in a sparsely populated area, having a low education level, being obese, and having a lower self-rated health. Public health initiatives to promote MSA in Croatia seem warranted, particularly in the population groups that are at the highest risk of not meeting the MSA guidelines. Given the very low prevalence of meeting MSA guidelines in Croatia, it seems reasonable to encourage public health agencies and other relevant governmental and non-governmental bodies to consider the development of comprehensive strategies for promoting MSA among adults.

## Figures and Tables

**Table 1:** Sample characteristics.

Population Group	*n*	%
All	4561	100.0
*Sex*		
Female	2401	52.6
Male	2160	47.4
*Age*		
18–24 years	458	10.1
25–39 years	1068	23.4
40–64 years	2122	46.5
65+ years	912	20.0
*Urbanisation level*		
Densely populated area	1164	25.5
Moderately populated area	1424	31.2
Sparsely populated area	1973	43.2
*Education level*		
Low	924	20.3
Medium	2597	56.9
High	979	21.5
*Smoking status*		
Non-smokers	3220	70.6
Smokers	1333	29.2
*Alcohol intake*		
Rarely or never	2970	65.1
2–3 days per month	437	9.6
1–2 days per week	430	9.4
3 or more days per week	702	15.4
*Body mass index*		
<18.5	87	1.9
18.5–24.9	1848	40.5
25–29.9	1801	39.5
≥30	825	18.1
*Self-perceived health*		
Very good	1080	23.7
Good	1640	36.0
Fair	1149	25.2
Bad or very bad	662	14.5

**Table 2:** Weighted ^a^ prevalence of meeting the muscle-strengthening activity (MSA) guidelines ^b^ across different population groups.

Population Group	% Meeting MSA Guidelines (95% CI ^c^)	***p*^d^**
All	8.0 (7.2, 8.7)	
*Sex*		<0.001
Female	5.4 (4.5, 6.4)	
Male	10.8 (9.6, 12.3)	
*Age*		<0.001
18–24 years	20.1 (16.4, 23.8)	
25–39 years	11.8 (9.9, 13.7)	
40–64 years	5.7 (4.7, 6.6)	
65+ years	2.7 (1.7, 3.8)	
*Urbanisation level*		<0.001
Densely populated area	11.4 (9.6, 13.2)	
Moderately populated area	8.0 (6.6, 9.4)	
Sparsely populated area	5.9 (4.9, 7.0)	
*Education level*		<0.001
High	14.1 (11.9, 16.3)	
Medium	7.9 (6.9, 9.0)	
Low	2.1 (1.1, 3.0)	
*Smoking status*		0.546
Non-smokers	7.8 (6.9, 8.7)	
Smokers	8.3 (6.8, 9.8)	
*Alcohol intake*		<0.001
Rarely or never	6.9 (6.0, 7.8)	
2–3 days per month	12.4 (9.3, 15.4)	
1–2 days per week	10.7 (7.8, 13.6)	
3 or more days per week	8.1 (6.1, 10.1)	
*Body mass index*		<0.001
<18.5	8.0 (2.3, 13.8)	
18.5–24.9	10.2 (8.8, 11.6)	
25–29.9	7.7 (6.5, 9.0)	
≥30	3.4 (2.2, 4.6)	
*Self-perceived health*		<0.001
Very good	14.7 (12.6, 16.8)	
Good	8.3 (7.0, 9.6)	
Fair	4.4 (3.2, 5.6)	
Bad or very bad	2.0 (0.9, 3.0)	

^a^ Sample weights taken from the European Health Interview Survey (EHIS wave 2; ^b^ Engaging in muscle-strengthening activities at least two times per week; ^c^ Ninety-five percent confidence interval; ^d^ *p*-value from the chi-square test.

**Table 3:** Adjusted odds ratios (OR) of meeting the muscle-strengthening activity (MSA) guidelines ^a^.

Population Group	Adjusted OR (95% CI ^b^)	***p*^c^**
All		
*Sex*		
Female	0.51 (0.39, 0.66)	<0.001
Male	ref	
*Age*		
18–24 years	ref	
25–39 years	0.49 (0.36, 0.68)	<0.001
40–64 years	0.31 (0.22, 0.44)	<0.001
65+ years	0.18 (0.11, 0.31)	<0.001
*Urbanisation level*		
Densely populated area	ref	
Moderately populated area	0.80 (0.60, 1.06)	0.118
Sparsely populated area	0.63 (0.48, 0.84)	0.002
*Education level*		
High	ref	
Medium	0.50 (0.39, 0.64)	<0.001
Low	0.23 (0.14, 0.38)	<0.001
*Smoking status*		
Non-smokers	ref	
Smokers	0.97 (0.76, 1.25)	0.824
*Alcohol intake*		
Rarely or never	ref	
2–3 days per month	1.25 (0.89, 1.76)	0.193
1–2 days per week	1.28 (0.89, 1.84)	0.187
3 or more days per week	1.27 (0.91, 1.79)	0.164
*Body mass index*		
< 18.5	0.80 (0.36, 1.77)	0.574
18.5–24.9	ref	
25–29.9	0.95 (0.73, 1.24)	0.708
≥ 30	0.55 (0.36, 0.86)	0.008
*Self-perceived health*		
Very good	ref	
Good	0.72 (0.55, 0.93)	0.013
Fair	0.60 (0.41, 0.86)	0.005
Bad or very bad	0.37 (0.20, 0.68)	0.002

^a^ Engaging in muscle-strengthening activities at least two times per week; ^b^ Ninety-five percent confidence interval; ^c^ *p*-value for adjusted odds ratio.

## Data Availability

Restrictions apply to the availability of these data. Data was obtained from Croatian Institute of Public Health with the permission of Croatian Institute of Public Health.
